# The Role of DaT-SPECT Imaging in the Evaluation of Progressive Supranuclear Palsy

**DOI:** 10.3390/life16060936

**Published:** 2026-06-01

**Authors:** Alexandros Giannakis, Konstantina Pakou, Spyridon Konitsiotis, Chrissa Sioka

**Affiliations:** 1 Department of Neurology, Faculty of Medicine, School of Health Sciences, University of Ioannina, University Campus, Stavrou Niarchou Av., 45500 Ioannina, Greece; kp1996@hotmail.com (K.P.); skonitso@uoi.gr (S.K.); 2 Department of Nuclear Medicine, Faculty of Medicine, School of Health Sciences, University of Ioannina, University Campus, Stavrou Niarchou Av., 45500 Ioannina, Greece; csioka@yahoo.com

**Keywords:** progressive supranuclear palsy, dopamine transporter, single-photon emission computed tomography, basal ganglia

## Abstract

Introduction: Progressive supranuclear palsy (PSP) is an atypical Parkinsonian disorder characterized by a range of clinical phenotypes, reflecting its multiple subtypes. As a result, accurate diagnosis during life remains challenging, underscoring the need for reliable biomarkers. The present narrative review aims to evaluate whether dopamine transporter single-photon emission computed tomography (DaT-SPECT) can serve as a biomarker in the assessment of PSP. Methods: The database search identified 31 original research articles relevant to our study objective. Of these, 17 studies included PSP patients and utilized DaT-SPECT as the sole molecular imaging modality; 9 studies combined DaT-SPECT with at least one additional molecular imaging technique; and 5 studies integrated DaT-SPECT with a laboratory-based biomarker of neurodegenerative disease. Results: DaT-SPECT appears to demonstrate low specificity and variable sensitivity for PSP across studies. Discussion: Combining DaT-SPECT with other diagnostic biomarkers, especially brain magnetic resonance imaging and other nuclear imaging modalities, may improve diagnostic accuracy, especially given its relatively low specificity for PSP. Nevertheless, these initially promising findings need to be validated in large, multicenter studies that include and clearly define multiple, autopsy-confirmed PSP subtypes.

## 1. Introduction

Progressive supranuclear palsy (PSP) is a rare, progressive neurodegenerative disorder that belongs to the group of atypical Parkinsonian syndromes. The condition is characterized by several key clinical features, including supranuclear vertical gaze palsy, postural instability with early falls, axial rigidity, and cognitive dysfunction. PSP presents with several clinical phenotypes. While the classic PSP–Richardson’s syndrome (PSP-RS) is the most common form, other subtypes include PSP with predominant Parkinsonism (PSP-P), progressive gait freezing (PSP-PGF), ocular motor dysfunction, postural instability (PSP-PI), and speech and language impairment (PSP-SL) [1].

The estimated prevalence ranges from 1.4 to 8.3 cases per 100,000 individuals. After the onset of symptoms, the median survival time is approximately 6.9 years, although this may vary among individuals [2]. Annual incidence increases with age, from 2/100,000 during the sixth decade of life to 15/100,000 during the ninth. Symptoms generally begin at 65 years of age. No difference appears to exist between sex, and apart from age, no risk factors appear to exist as well [3,4]. PSP is considered a sporadic disease, although some mutations appear to increase the risk for PSP, including mutations in the *MAPT*, *GRN*, *FUS*, and *TARDBP* genes [3,5,6].

Neuropathologically, the hallmark of the disease consists of aggregates of the microtubule-associated protein tau (MAPT), which accumulate within pathologically altered tufted astrocytes. These aggregates are also found within oligodendrocytic coils or as neurofibrillary tangles [7]. The physiological role of the tau protein within cells is to stabilize microtubules by binding to polymerized tubulin [8]. This function is of particular importance in neurons, where microtubule stability is a prerequisite for axonal stability [9]. The tau protein is encoded by the *MAPT* gene, located on chromosome 17. Alternative splicing of the *MAPT* gene gives rise to six isoforms of the tau protein. The tau protein contains specific microtubule-binding domains within its molecule. Depending on whether it carries 3 or 4 repeats of the binding domain (which is determined by the inclusion or exclusion of exon 10 during splicing), the tau protein is classified into 3-repeats and 4-repeats (4R). PSP is classified as a primary 4R tauopathy, meaning that the accumulation of 4R tau aggregates represents the core, driving neuropathology rather than a secondary consequence of another neurodegenerative process within the brain [10,11].

The clinical diagnosis of PSP remains challenging, especially in the early stages of the disease. Early symptoms often overlap with those of Parkinson’s disease (PD) and atypical Parkinsonian disorders (APDs), which can lead to misdiagnosis or delayed recognition [12]. The latest PSP criteria incorporate clinical features, neuropathological findings, therapeutic response, and neuroimaging results. A definitive diagnosis requires neuropathological confirmation, which can only be obtained through post-mortem examination, making it impossible to establish with certainty during the patient’s lifetime [1].

Following the publication of the Movement Disorders Society (MDS) revised criteria, an autopsy-confirmed study assessed their sensitivity and specificity. Results showed that the MDS criteria demonstrated improved sensitivity (87.9%), although specificity was somewhat reduced (82%) compared with the previous National Institute of Neurological Disorders and Stroke (NINDS) criteria [1,12,13].

Nevertheless, the need for a highly sensitive and specific biomarker for PSP remains critical. Given the current lack of disease-modifying therapies, accurate detection of PSP in its earliest stages—where therapeutic interventions are most likely to be effective—is crucial for enrolling patients into clinical trials. Furthermore, a biomarker capable of monitoring disease progression and correlating with clinical symptoms would be invaluable for elucidating the underlying pathological mechanisms [14,15].

Dopamine transporter single-photon emission computed tomography (DaT-SPECT) uses a radiopharmaceutical agent to visualize the dopamine transporter (DaT) in the striatum, serving as an in vivo marker of nigrostriatal pathway integrity. The technique enables both visual and semiquantitative assessment of striatal DaT binding, including the calculation of the specific binding ratio (SBR) relative to reference regions. Reduced striatal tracer uptake reflects the loss of presynaptic dopaminergic terminals, which is a characteristic feature of degenerative Parkinsonian syndromes [16].

The use of DaT-SPECT in PSP is justified by the involvement of the nigrostriatal dopaminergic system, as PSP is associated with presynaptic dopaminergic degeneration and reduced striatal dopamine transporter availability [17]. While DaT-SPECT can reliably demonstrate dopaminergic loss and distinguish PD and APDs (including PSP) from essential tremor (ET), drug-induced Parkinsonism (DIP) and healthy individuals, its major limitation lies in the poor differentiation between PSP, PD and APDs, which often exhibit similar patterns of nigrostriatal impairment [18,19].

This limitation has prompted increasing interest in identifying disease-specific imaging patterns and applying advanced analytical methods to improve differential diagnosis [19]. The purpose of this narrative review is to evaluate the clinical utility of DaT-SPECT imaging in patients with PSP, including its diagnostic performance, its capacity to monitor disease progression, and its correlation with clinical symptoms.

## 2. Methods

### 2.1. Source Search

**Figure 1:**
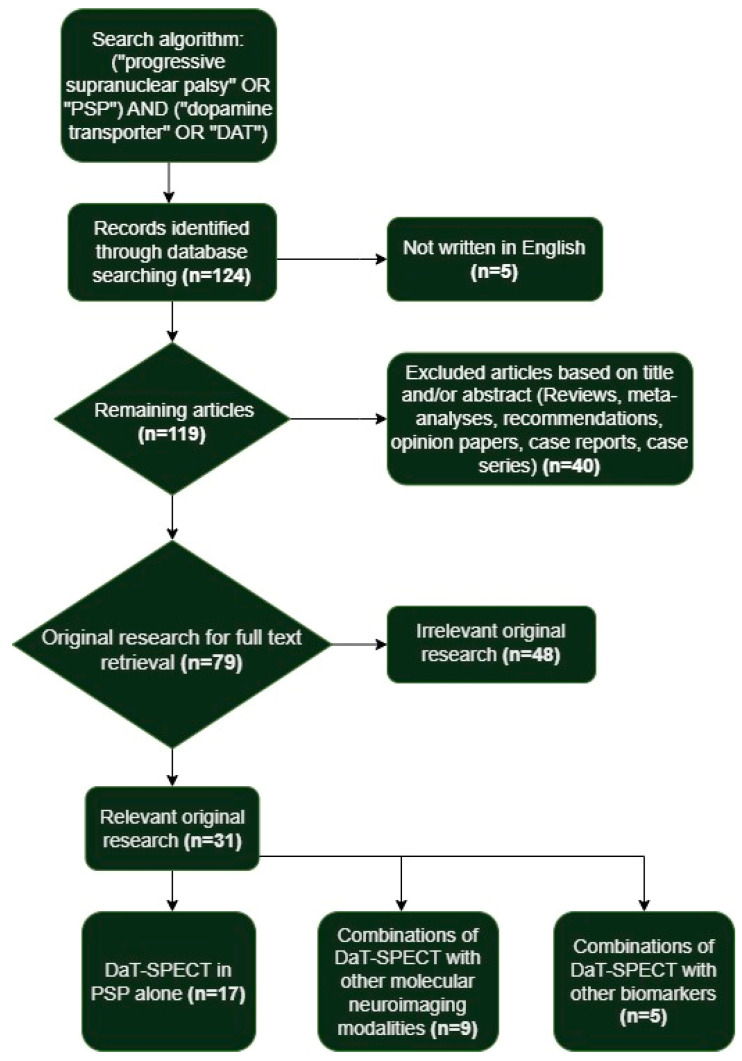
Study selection process.

The study selection process is depicted in Figure 1. The study selection process was informed by the Scale for the Assessment of Narrative Review Articles (SANRA) [20]. The PubMed database was searched from inception until 28 February 2026 using the following algorithm: (“progressive supranuclear palsy” OR “PSP”) AND (“dopamine transporter” OR “DaT”).

### 2.2. Quality Assessment of Studies

Studies were included if they met the following criteria:

- Articles published in English with available full text.
- Original research papers.
- Included at least one cohort or treatment arm consisting of patients with PSP, diagnosed based on the validated clinical criteria applicable at the time of the study.
- Utilized DaT-SPECT imaging within the PSP cohort for the study.

Notably, given the methodological heterogeneity among studies, including differences in radiotracer preparation and image acquisition protocols, we did not apply highly specific methodological inclusion criteria, such as image acquisition timing or the exclusive use of visual versus semi-quantitative analysis. This approach was intentionally adopted to maximize the number of studies included in the current review and to more comprehensively reflect the methodological heterogeneity present in the existing literature.

The search yielded 124 results in total. Of these, 5 articles were excluded because they were not written in English. The remaining 119 articles had their titles and abstracts screened, and 40 articles were excluded as they were not original research (including reviews, meta-analyses, recommendations, opinion papers, case reports, and case series). Subsequently, 80 original research articles were obtained for full-text retrieval.

Finally, 31 articles met the criteria for inclusion in our review. It should also be noted that 52 additional studies are referenced in this review for theoretical background, discussion, and interpretation of the obtained results.

### 2.3. Data Conceptualization

While several studies utilized DaT-SPECT as the standalone nuclear imaging modality in PSP patients, others combined it with complementary nuclear medicine techniques or laboratory biomarkers. Therefore, to better synthesize and understand the heterogeneous data, we categorized the selected articles into three distinct groups:

- studies including at least one PSP patient group that underwent DaT-SPECT as the sole molecular imaging modality (17 studies);
- studies including at least one PSP patient group that underwent DaT-SPECT in combination with at least one additional molecular imaging modality, but without any laboratory-based neurodegenerative disease biomarkers (9 studies);
- studies including at least one PSP patient group that underwent DaT-SPECT together with at least one laboratory-based neurodegenerative disease biomarker, regardless of whether additional nuclear imaging modalities besides DaT-SPECT were also used (5 studies).

## 3. Results

### 3.1. DaT-SPECT as the Sole Imaging Modality in Patients with PSP

Table 1 presents the key findings of studies employing DaT-SPECT alone in PSP patients.

The Parkinson Study Group enrolled patients with PSP, PD, ET, and healthy controls to compare basal ganglia uptake of ^123^I 2β-carboxymethoxy-3β-(4-iodophenyl)tropane (^123^I-β-CIT). They found that patients with Parkinsonian syndromes exhibited reduced dopamine uptake in both the caudate nucleus and the putamen compared with patients with ET and healthy controls. Moreover, visual interpretation of the scans demonstrated high sensitivity and specificity for distinguishing PD and PSP from the other study groups. When comparing PSP and PD, patients with PD showed lower uptake in the caudate nucleus [21]. In contrast, when Seppi et al. utilized the same radiotracer in patients with PSP, PD, MSA with predominant Parkinsonism (MSA-P), and healthy controls, they found that all patient groups exhibited reduced uptake in the basal ganglia compared with controls. Interestingly, both MSA-P and PSP showed reduced brainstem uptake compared with PD, with decreases in midbrain uptake being able to discriminate PD from APDs with high accuracy. Nevertheless, this measure was unable to effectively differentiate PSP from MSA-P [22]. On the other hand, when Goebel et al. applied a computer-assisted algorithm to similar patient groups, they achieved higher diagnostic accuracy compared with visual assessment alone. Diagnostic accuracy was particularly high when PSP and MSA-P were analyzed as a combined group [23].

Im et al. utilized ^123^I N-(3-iodopropen-2-yl)-2β-carbomethoxy-3β-(4-chlorophenyl) tropane (^123^I-IPT) in patients with PSP, PD, and healthy controls. After dividing the basal ganglia into four regions of interest (ROIs), they found that patterns of uptake reduction differed between the two patient groups. Specifically, the PSP group showed more evenly and widely distributed reductions across all ROIs, whereas in patients with PD the reduction in uptake followed a more rostro-caudal pattern, with the putamen being more severely affected than the head of the caudate. The greatest differences in radiotracer uptake between PSP and PD were observed in the second ROI, a transitional region between the head of the caudate and the putamen [24].

The majority of studies utilized ^123^I N-ω-fluoropropyl-2β-carbomethoxy-3β-(4-iodophenyl) nortropane (^123^I-FP-CIT) as a DaT radiotracer. Antonini et al. compared patients with PSP to those with PD, MSA-P, and healthy controls; they found reduced dopamine uptake in the PSP group compared with both the PD and MSA-P groups using ^123^I-FP-CIT. Moreover, the PD group showed relatively lower uptake in the putamen compared with the caudate, whereas in the PSP group, the reduction was more evenly distributed between the caudate and putamen. This pattern may suggest a more diffuse dopaminergic degeneration in PSP [25]. Likewise, when Filippi et al. utilized the same radiotracer in patients with PSP, PD, and controls, they found reduced uptake in the caudate, putamen, and basal ganglia overall in both PSP and PD patients compared with controls. Moreover, the reduction in uptake was greater in all ROIs in PSP than in PD. Lastly, uptake asymmetry was more pronounced in the PD group than in the PSP group, with greater decrements observed in the hemisphere contralateral to the clinically more affected side [16].

Roselli et al. exploited the high affinity of ^123^I-FP-CIT for the serotonin transporter, in addition to the DaT, to investigate tracer uptake in the midbrain among patients with PSP, PD, dementia with Lewy bodies (DLB), ET, and healthy controls. They observed slight reductions—along with considerable interindividual variability—in the PD group compared with ET patients and controls. In contrast, midbrain uptake was markedly reduced in PSP and nearly absent in DLB. These findings suggest that DaT-SPECT imaging may have potential applications beyond its original purpose [26]. ^123^I-FP-CIT was used in a similar manner by Joling et al. in patients with PSP, PD, MSA-P, and MSA-cerebellar type (MSA-C). In addition to reduced tracer uptake in the caudate nucleus in PSP compared with PD and MSA-C, the authors also reported reduced uptake in the hypothalamus—a region where the serotonin transporter is also expressed—in PSP compared with MSA-C. Conversely, uptake in the posterior putamen was lower in all patient groups compared with MSA-C. Furthermore, voxel-based analysis demonstrated reduced hypothalamic tracer uptake in PSP and MSA-P compared with PD and MSA-C [27].

Badoud et al. studied a large patient cohort comprising more than 300 patients with PD who underwent ^123^I-FP-CIT DaT-SPECT imaging. Patients with PSP, MSA, and CBD were also included in the analysis. Their results showed that tracer uptake in the head of the caudate nucleus was more severely reduced in patients with PSP and MSA compared with those with PD and CBD. However, the diagnostic accuracy of this method proved to be limited, particularly in distinguishing PSP from MSA, PSP from CBD, and PD from the combined group of APDs [28]. In another study that enrolled patients with a wide range of causes of Parkinsonism—including PSP, PD, CBD, MSA, and DLB, as well as vascular Parkinsonism (VaP), ET, normal pressure hydrocephalus (NPH), and DIP—the authors found that both visual and semi-quantitative analysis of ^123^I-FP-CIT uptake on DaT-SPECT yielded similarly satisfactory sensitivity when differentiating neurodegenerative Parkinsonian syndromes, including PSP, from non-neurodegenerative causes of Parkinsonism. Notably, sensitivity was even higher when visual and semi-quantitative assessments were combined [19]. However, the results reported by Sakamoto et al. were different. The researchers utilized ^123^I-FP-CIT and calculated the SBR of the basal ganglia in patients with PSP, PD, MSA, and CBD to investigate its ability to discriminate PSP from other Parkinsonian syndromes. Additionally, they calculated the magnetic resonance parkinsonism index (MRPI), an index based on brain magnetic resonance imaging (MRI) that is highly sensitive and specific for PSP. While MRPI achieved very high sensitivity, specificity, and accuracy for diagnosing PSP compared with the other study groups, SBR showed high sensitivity but very low specificity for PSP [29]. Furthermore, when Seckin et al. utilized DaT-SPECT to compare patients with primary progressive apraxia of speech (PPAOS)—a condition that eventually progresses to either PSP or CBD in the majority of cases—with patients with PSP and CBD, they found similar, symmetric patterns of decrements in radiotracer uptake in the basal ganglia across the three study groups. The only exception was a greater decrement in the posterior putamen in the PSP group [30].

In the study conducted by Shigekiyo et al., the average SBR and SBR laterality were measured in patients with PSP, PD, MSA-P, and controls. Notably, PD patients were further categorized based on their Hoehn and Yahr scale (HY) stage. The authors found that both SBR laterality and average SBR were higher in PD patients at HY stage I (early stage) compared with those with PSP. However, other SBR measures were similar between PSP and the other study groups, including patients with more advanced-stage PD [31]. Multiple ^123^I-FP-CIT metrics, including mean SBR, SBR laterality, asymmetry index, and the caudate/putamen ratio, were utilized by Constantinides to investigate the discriminating ability of DaT-SPECT. The patient groups encompassed individuals with PSP, PD, MSA-P, and CBD, as well as healthy controls. Specifically for PSP, the mean striatal SBR was able to discriminate between PSP and CBD with modest sensitivity and good specificity. However, uptake metrics were similar between the PSP, PD, and MSA-P groups. [32]. In a study primarily focused on differentiating PD dementia (PDD) from DLB using DaT-SPECT, Ishizawa et al. found reduced tracer uptake in the posterior striatum in patients with PD and PDD. However, tracer uptake did not differ among patients with PSP, DLB, and MSA-P. Even when Kawazoe et al. combined the SBR from DaT-SPECT with neuromelanin-related contrast of the substantia nigra on MRI in patients with PSP, MSA-P, and CBD, no significant differences were observed between the PSP and CBD groups across any of the study parameters. Moreover, no correlations between SBR and MRI findings were identified among the three study groups [34].

Lastly, in one of the few studies that included autopsy-confirmed cases, Hastings et al. analyzed a retrospective cohort of patients with PSP, PD, MSA, and CBD who underwent DaT-SPECT. The authors reported a high sensitivity of DaT-SPECT for detecting PSP. Sensitivity was similarly high in PD and MSA, but lower in CBD. However, specificity was low across all groups, including PSP, resulting in a mediocre negative predictive value (NPV). Notably, abnormal DaT-SPECT findings were more frequent in the classic PSP-RS compared with other PSP subtypes [35].

### 3.2. DaT-SPECT Combined with Other Molecular Imaging in PSP Patients

The main findings of studies that utilized DaT-SPECT combined with another molecular imaging modality are summarized in Table 2.

Combinations of DaT-SPECT with other molecular imaging modalities can be further classified into three main categories:

- studies combining DaT-SPECT with postsynaptic D2 dopamine receptor imaging;
- studies combining DaT-SPECT with brain perfusion SPECT;
- studies combining DaT-SPECT with cardiac ^123^I metaiodobenzylguanidine (^123^I-MIBG) imaging.

Postsynaptic D2 receptor imaging with ^123^I-iodobenzofuran (^123^I-IBF) and DaT-SPECT was performed by Kim et al. in patients with PSP, PD, MSA, and healthy controls. DaT uptake was reduced in the posterior putamen in all patient groups but failed to differentiate among them. In contrast, D2 receptor uptake was higher in the caudate PD and PSP than in MSA [36]. Plotkin applied DaT-SPECT and ^123^I-iodobenzamide (^123^I-IBZM) for postsynaptic D2 receptor single-photon emission computed tomography (SPECT) imaging in patients with PSP, PD, MSA, CBD, DLB, and ET. The majority of patients with a Parkinsonian syndrome had abnormal DaT imaging. D2 postsynaptic tracer uptake was normal in the majority of patients with CBD and in all patients with ET and PD, whereas it was pathological in the majority of PSP cases and in some MSA patients. These findings suggest that postsynaptic D2 receptor imaging may assist in the diagnostic differentiation between PSP and ET or PD, but is less helpful for distinguishing PSP from MSA and CBD [37]. Lin et al. used two radiotracers: [2-[[2-[[[3-(4-chlorophenyl)-8-methyl-8-azabicyclo [3,2,1]oct-2-yl]methyl](2-mercaptoethyl)amino]ethyl]amino]ethanethiolato(3-)-N2,N20,S2,S20]oxo [1R-(exo-exo)])-[99mTc] dopamine transporter (^99m^Tc-TRODAT-1) for dopamine transporter imaging with DaT-SPECT and ^123^I-IBZM. They enrolled patients with PSP, PD, and normal controls. Notably, patients with PSP were further subdivided into PSP-RS and PSP-P subtypes. Higher, although not statistically significant, mean DaT uptake was observed in the PSP-RS group compared with the PSP-P group. An increased caudate-to-putamen uptake ratio was found in PD, in contrast to both PSP subtypes, where a decreased ratio was observed. On the other hand, IBZM uptake was reduced in the PSP-RS group, whereas only mild changes were observed in the PSP-P and PD groups compared with controls [38]. Similarly, Jakobson et al. utilized both DaT-SPECT and ^123^I-IBZM imaging in patients with PSP, PD, MSA, and healthy controls. Notably, the majority of PD patients and some patients with PSP and MSA repeated the examination after three years. Interesting findings emerged from this longitudinal follow-up. Although PSP exhibited lower striatal DaT uptake during the initial examination compared with MSA, uptake levels were similar after three years. Moreover, MSA demonstrated higher ^123^I-IBZM uptake compared with PSP during the first evaluation and compared with all study groups during the second evaluation [39].

Another combination of molecular imaging that has been investigated is that of DaT-SPECT and brain perfusion SPECT. Van Laere et al. combined DaT-SPECT with ^99m^Tc-ethylcysteinate dimer (^99m^Tc-ECD) brain perfusion SPECT. The study groups included patients with PSP, PD, MSA, DLB, and ET. Similar to previous studies, the PSP group exhibited lower DaT uptake in the caudate nucleus bilaterally, similar to that observed in MSA and DLB. Brain perfusion SPECT revealed a characteristic hypoperfusion pattern in PSP, primarily involving the midbrain, prefrontal cortex, anterior cingulate cortex, thalami, and caudate nuclei. The anterior-to-posterior putamen ratio of the left side differed significantly between PSP and PD and MSA patients. However, DaT-SPECT and brain perfusion SPECT alone demonstrated low diagnostic accuracy in discriminating between Parkinsonian syndromes. Only when combined did they achieve improved diagnostic accuracy [18]. ^123^I-iodoamphetamine (^123^I-IMP) brain perfusion SPECT in combination with DaT-SPECT was utilized by Takaya et al. Contrary to Van Laere et al., data from both imaging modalities were combined with ROIs derived from anatomical MRI. The investigators enrolled patients with PSP, PD, MSA, DLB, and CBD. The combined analysis yielded very high diagnostic accuracy in discriminating PSP, MSA, and CBD from PD and DLB. Moreover, similar accuracy was achieved in discriminating PSP from MSA and CBD [40]. Nakano et al. also achieved good diagnostic accuracy using ^123^I-IMP brain perfusion SPECT combined with DaT-SPECT in patients with PSP, PD, MSA, and CBD. Interestingly, specific combinations of hypoperfusion patterns and reduced DaT binding were identified for each disorder, including PSP, PD, and CBD. Specifically, for the PSP-RS subtype, ipsilateral SBR was positively correlated with perfusion of the caudate nucleus bilaterally, as well as with perfusion of the ipsilateral putamen and ipsilateral prefrontal cortex [41].

DaT-SPECT in PSP has also been combined with other nuclear imaging modalities. Some studies have investigated cardiac ^123^I-metaiodobenzylguanidine (^123^I-MIBG) scintigraphy alongside dopaminergic imaging. Niimi et al. studied a small cohort of patients with PSP, PD, MSA, CBD, and unclassified Parkinsonism. All patients first underwent cardiac ^123^I-MIBG scintigraphy, which showed normal results. Subsequently, they underwent DaT-SPECT imaging. The results demonstrated a lower striatal asymmetry index (SAI) in patients with PD compared with those with APDs. However, the SBRs were similar across all study groups [44]. Likewise, Iwabuchi et al. enrolled a larger cohort of patients with PSP, PD, MSA, CBD, DLB, and a control group without Parkinsonism. Both DaT-SPECT and cardiac ^123^I-MIBG scintigraphy were performed, and multiple parameters from both imaging modalities were combined to investigate their diagnostic accuracy across the study groups. Specifically for PSP, very high specificity and NPV were observed; however, sensitivity was low. The parameters that contributed most to discriminating between the study groups were the SBR and the putamen-to-caudate ratio for DaT-SPECT, as well as the delayed heart-to-mediastinum ratio and the cardiac washout rate for ^123^I-MIBG scintigraphy [43].

### 3.3. DaT-SPECT Combined with Laboratory Biomarkers in PSP

Apart from MRI and other nuclear imaging modalities, DaT-SPECT has also been combined with several cerebrospinal fluid (CSF), serum, and electrophysiological biomarkers for various purposes. Table 3 summarizes the principal findings from studies that have integrated DaT-SPECT imaging with laboratory biomarkers in patients with PSP.

Suzuki et al. utilized a heterogeneous study cohort comprising a wide spectrum of neurodegenerative diseases, including PSP, PD, MSA, CBD, and DLB, as well as non-Parkinsonian syndromes such as Alzheimer’s disease (AD), frontotemporal dementia (FTD), and amyotrophic lateral sclerosis (ALS). Their main aim was to investigate possible correlations between various clinical parameters and serum insulin-like growth factor 1 (IGF-1) levels among the aforementioned diseases. For the subset of patients with Parkinsonian syndromes, DaT-SPECT imaging was also performed. A positive correlation was observed between serum IGF-1 levels and the SBR in patients with PD. However, this correlation was not found in APDs, including PSP. Moreover, both IGF-1 levels and SBR values were similar across the study groups [45].

Contrary to Suzuki et al., who assessed a serum biomarker, Diekämper et al. combined DaT-SPECT imaging with various CSF biomarkers. Some of these, such as α-synuclein, are relatively specific to certain neurodegenerative diseases, whereas others—including neurofilament light chain (NfL) and phosphorylated neurofilament heavy chain (pNfH)—serve as non-specific indicators of neuronal structural integrity. The study included patients with PSP, PD, DLB, and CBD, as well as a group of AD patients who were used as a control group for DaT-SPECT. In addition, a separate healthy control group was included for comparison of CSF biomarkers. A strong negative correlation was observed between bilateral caudate nucleus and putamen uptake and CSF levels of NfL and pNfH across all Parkinsonian disorder groups. However, no significant differences were found between these patient groups. Moreover, CSF levels of α-synuclein, progranulin, and total tau protein did not correlate with DaT-SPECT uptake.

Goto et al. compared DaT-SPECT findings in patients with PSP, PD, MSA, and CBD, and investigated potential correlations between DaT uptake and CSF homovanillic acid (HVA) levels. An AD group was included for comparison. Significantly lower CSF HVA levels were observed in patients with PSP, PD, and MSA compared to those with AD. Additionally, the SBR in DaT-SPECT was significantly lower in PSP patients compared to PD patients. Interestingly, a positive correlation was found between CSF HVA levels and SBR in DaT-SPECT in both the PSP and PD groups.

The coexistence of PSP and normal pressure hydrocephalus (NPH) has been investigated by Shimada et al. In this retrospective study, the researchers recruited patients with NPH alone as well as patients with coexisting PSP and NPH. The latter group included three PSP subtypes: PSP-RS, PSP-P, and PSP-PGF. All patients underwent DaT-SPECT and brain MRI, while CSF α-synuclein levels were also measured in both groups. All patients tested negative for CSF α-synuclein. The average SBR was reduced in the PSP group compared to the NPH-only group, along with corresponding alterations in the midbrain tegmentum. Moreover, asymmetrically reduced DaT uptake was observed in the PSP group. Notably, 8 out of 15 patients with coexisting PSP and NPH underwent lumboperitoneal shunt placement, which resulted in clinical improvement one year after surgery [48].

Lastly, Ozawa et al. conducted a multimodal assessment combining DaT-SPECT, cardiac ^123^I-MIBG scintigraphy, and SSR testing in patients with PSP, PD, and MSA. Their findings demonstrated that SSR amplitude showed a significant correlation with both striatal dopaminergic dysfunction, as measured by DaT-SPECT, and cardiac sympathetic denervation, as assessed by ^123^I-MIBG uptake—but this relationship was observed exclusively in the PD cohort. No such correlations were identified in patients with PSP or MSA [49].

### 3.4. DaT-SPECT in PSP-RS

As previously noted, it is the most common phenotypic subtype of PSP. However, as demonstrated in the preceding subsections, only four studies have evaluated PSP-RS as an independent subgroup for direct comparison with either alternative PSP phenotypes or other atypical Parkinsonian syndromes [35,38,41,48]. In the sole study that specifically evaluated pathologically confirmed cases of PSP-RS, the specificity of DaT-SPECT remained consistently low across all cohorts, including the PSP-RS, non-RS PSP subtypes, and other Parkinsonian disorders [35]. While that study observed a slightly more frequent DaT uptake abnormality in PSP-RS compared to other phenotypes, contrasting results were reported by Lin et al. They found that DaT uptake was actually higher in the PSP-RS subgroup than in the PSP-P cohort; notably, however, postsynaptic ^123^IBZM binding was differentially reduced in the PSP-RS group compared to both the PSP-P and PD cohorts [38].

Further phenotypic differences emerged in the remaining two studies. Nakano et al. demonstrated that the PSP-RS subtype exhibited a correlation between the SBR and ipsilateral perfusion of the caudate, putamen, and prefrontal cortex, as well as the contralateral caudate, a finding not replicated in the PSP-PGF, PSP-PI, or PSP-SL subgroups [41]. Finally, Shimada et al. reported asymmetrically reduced DaT uptake across all evaluated cohorts, including the PSP-RS, PSP-P, and PSP-PGF subtypes [48].

## 4. Discussion

The majority of studies have used DaT-SPECT imaging as a technique to differentiate PSP from PD and other APDs. In some studies, additional comparison groups, including ET, NPH, VaP, DIP, and healthy controls, were also evaluated. Overall, the evidence suggests that DaT-SPECT can effectively distinguish PD and APDs (i.e., PSP, CBD, MSA, and DLB), from ET, DIP, and controls [19,21].

Interestingly, findings from some studies suggest that DaT-SPECT may also be utilized as a biomarker of disease progression and for assessing correlations among disorders within a similar spectrum. For instance, similar DaT uptake patterns have been observed in patients with PPAOS and CBD. In contrast, slightly reduced uptake in the posterior putamen has been reported in PSP, which may indicate a distinct pattern of disease progression among these conditions despite their significant phenotypic and pathological overlap [1,30,50]. In addition, DaT-SPECT has also been linked to levels of laboratory biomarkers associated with neurodegeneration, such as NfL and HVA in the CSF, suggesting that it may serve as a connective link between pathological progression and loss of functionality in neurodegenerative Parkinsonian disorders [46,47].

Most importantly, within a disease spectrum where multiple conditions may coexist [51], DaT-SPECT, when combined with other imaging modalities such as MRI, appears to differentiate patients with PSP who also have specific syndromes that may influence treatment planning and disease progression. For example, patients with coexistent NPH and PSP may show improvement following lumboperitoneal shunting, in contrast to those with PSP alone [48]. This suggests that DaT-SPECT may also serve as a biomarker for selective and individualized treatment management.

However, ambiguous results emerge from studies attempting to discriminate between PSP, PD, and APDs. While grouping APDs, including PSP, and comparing them to PD may provide useful insight for distinguishing PD [23], DaT-SPECT has generally shown variable sensitivity and specificity in differentiating PSP from other APDs, such as MSA and CBD [29,32]. Even when specific patterns of DaT uptake are used to differentiate between Parkinsonian disorders, the results remain inconsistent. Some studies identify patterns that differentiate PSP and MSA from PD [23,27], and others separate MSA and PD from PSP [25,28]. These discrepancies make it difficult to establish a consistent and reliable DaT uptake pattern capable of effectively distinguishing PSP from other neurodegenerative Parkinsonian disorders. Most importantly, even these seemingly distinct patterns of DaT uptake between PSP and other Parkinsonian syndromes may be present only in the early stages of the neurodegenerative Parkinsonian syndromes, but tend to diminish as these diseases progress, eventually becoming nearly identical and difficult to differentiate [31,39]. This variability likely reflects differences in study design, patient selection, disease stage, radiotracer type, image acquisition and reconstruction protocols, and methods of image interpretation. In particular, discrepancies between visual and semiquantitative analyses, as well as heterogeneity in clinical diagnostic criteria and lack of neuropathological confirmation in many cohorts, may have contributed to inconsistent findings. Disease stage is also an important factor, since early PSP and MSA-P frequently demonstrate overlapping patterns of nigrostriatal degeneration that reduce diagnostic discrimination [34].

Issues also arise regarding the methodologies of the included studies. First, the small sample size represents a major limitation, as the majority of studies enrolled fewer than one hundred patients. Given the low incidence of PSP and other APDs, it is unlikely that a single center with access to DaT-SPECT could recruit larger cohorts. Therefore, this limitation can likely only be addressed through the design of large, multicenter studies conducted by specialized clinics in movement disorders and/or neurodegenerative diseases, using standardized and shared protocols.

Another methodological drawback is that only a limited number of studies specify the PSP subtypes represented within their patient populations [38,48]. Considering the substantial phenotypic variability of PSP, and the fact that each subtype may pose distinct diagnostic challenges in relation to different neurodegenerative conditions (e.g., PSP-RS and PSP-P versus PD, PSP-PGF versus NPH), the absence of clearly defined subgroups may limit the validity of the findings when PSP is broadly compared with other Parkinsonian syndromes [1,52,53,54]. Nevertheless, when PSP-RS and PSP-P were analyzed as separate subgroups, differences in DaT uptake patterns were observed between them. However, these differences did not reach statistical significance, a limitation that may be attributable to the small sample size and could potentially be addressed in studies with larger cohorts [38].

It should also be noted that dopamine transporter positron emission tomography (DaT-PET), a newer nuclear imaging modality with a variety of available radiotracers, has been utilized in PSP and has provided valuable insights into disease subtypes and their differentiation from other neurodegenerative disorders [55,56,57,58,59]. Similar to DaT-SPECT, DaT-PET has been combined with other nuclear imaging techniques to further investigate the pathophysiological processes underlying disease progression in PSP [60,61,62,63]. Furthermore, several studies have reported higher sensitivity, specificity, and negative predictive value compared with DaT-SPECT in differentiating neurodegenerative Parkinsonian syndromes [64,65,66,67,68]. Consequently, it has been suggested that DaT-SPECT may be more suitable for the initial distinction between neurodegenerative and non-neurodegenerative Parkinsonism, whereas DaT-PET may offer additional value in the further differentiation of neurodegenerative conditions [69]. Nevertheless, despite its advantages, DaT-PET remains less widely available and more costly than DaT-SPECT [69], and it does not appear to reliably differentiate between PSP subtypes [70].

However, combining DaT-SPECT with other nuclear imaging modalities may improve diagnostic accuracy. Promising results have been reported with the combined use of ^123^I-MIBG and DaT-SPECT, demonstrating high specificity for PSP [43]. Additionally, the combination of DaT-SPECT with brain perfusion SPECT greatly enhances diagnostic accuracy for PSP in direct comparisons with other Parkinsonian disorders, including DLB, MSA, CBD, and PD [40]. Furthermore, postsynaptic D2 basal ganglia imaging may aid in differentiating PSP from MSA, once both have been distinguished from PD based on DaT-SPECT findings [36]. Nevertheless, combining nuclear imaging modalities entails additional, time-consuming procedures that require prolonged patient cooperation—often challenging in individuals with APDs due to impaired mobility and overall condition [71]. On the other hand, brain MRI, a modality routinely performed in the evaluation of neurodegenerative syndromes with dementia such as PSP, according to multiple guidelines [72,73,74], may be effectively combined with DaT-SPECT. Interestingly, specific MRI metrics, such as the MRPI, which demonstrate high specificity for PSP, when used alongside DaT-SPECT, which appears to offer high sensitivity, may substantially improve overall diagnostic accuracy in correctly identifying PSP [29].

Nevertheless, in our opinion, the principal limitation of nearly all included studies is the lack of autopsy-confirmed diagnoses in PSP cases. With the exception of the retrospective study by Hastings et al., and only two PSP cases in the study by Goto et al. [35,47]. All other studies relied solely on clinical criteria for patient recruitment. This represents a critical issue, as the very motivation for identifying reliable diagnostic biomarkers in PSP stems from the inherent challenges and limitations of clinical diagnosis [75]. Although the most recent diagnostic criteria have improved sensitivity, this has come at the expense of a slight reduction in specificity compared to earlier criteria, which, while highly specific, demonstrated unacceptably low sensitivity [1,12,53,76]. Consequently, in the absence of neuropathological confirmation, cases clinically diagnosed as PSP may in fact represent other neurodegenerative disorders, thereby compromising the validity of diagnostic performance measures and the overall evaluation of DaT-SPECT in these populations. Furthermore, accumulating evidence suggests that multiple pathologies frequently coexist within the same patient [77,78,79]. Indeed, some researchers propose that co-pathology may be the rule rather than the exception in neurodegenerative diseases [80]. Therefore, autopsy confirmation is essential not only to verify that patients evaluated with DaT-SPECT truly had PSP, but also to identify the presence of concomitant pathologies that may influence both the clinical phenotype and imaging findings.

The regions where pathological 4R aggregates are most frequently localized in PSP are primarily the midbrain, particularly the rostral interstitial nucleus of the medial longitudinal fasciculus, which serves as the control center for vertical eye movements, the superior colliculi, the substantia nigra, the locus coeruleus, the periaqueductal gray matter, the inferior olivary nucleus, and the basis pontis [7].

While DaT-SPECT is undeniably robust at confirming a core neurodegenerative Parkinsonian process, this review highlights its major diagnostic caveat: the inability of a standard qualitative visual binary read (normal vs. abnormal) to differentiate PSP from other causes of neurodegenerative Parkinsonism. Because the underlying 4R tau molecular pathology in PSP drives aggressive, topographically extensive cell death within the midbrain, the resulting presynaptic denervation mirrors that of other neurodegenerative Parkinsonian disorders on a standard scan [81]. However, emerging quantitative and semi-quantitative metrics (e.g., analyzing the putamen-to-caudate ratio) demonstrate diagnostic promise.

Regardless, in the study by Hastings et al., DaT-SPECT performed during life in ten autopsy-confirmed PSP cases showed pathological findings with high sensitivity, while specificity was low [35]. Nevertheless, since the clinical diagnostic criteria for PSP lack sensitivity but have high specificity [1,12], combining them with DaT-SPECT could potentially increase diagnostic accuracy. Alternatively, combining DaT-SPECT with MRPI derived from MRI, which also demonstrates excellent specificity [29], could further improve diagnostic accuracy, particularly in clinically complex cases. However, to validate these assumptions, large multicenter studies of autopsy-confirmed cases are needed. These studies should include and clearly define the various PSP subtypes and incorporate both brain MRI and DaT-SPECT.

Hence, to advance the clinical utility of DaT-SPECT and accelerate therapeutic development in PSP, future prospective trials must pivot from descriptive studies toward standardized, multi-center protocols. Based on the studies included in the current review, we propose optimal multimodal combinations and diagnostic sequencing. Relying on a single imaging modality often lacks the specificity required to differentiate PSP from other APDs early in the disease course. Future trial designs should employ a standardized, multimodal hierarchy. Frontline assessment should combine presynaptic dopaminergic imaging (DaT-SPECT) with high-resolution structural MRI to evaluate midbrain-to-pons ratios, such as the MRPI [29].

A further challenge in the interpretation of DaT-SPECT findings relates to the variability between visual and semi-quantitative analytical approaches. Future standardization efforts should focus on harmonizing acquisition and processing protocols, establishing validated normative databases and universally accepted quantitative thresholds, and developing consensus interpretation guidelines. Integrating semi-quantitative metrics with expert visual assessment may ultimately represent the most reliable strategy for reducing discrepancies between approaches and improving the consistency of DaT-SPECT implementation across clinical and research settings.

Subsequently, in cases with borderline DaT-SPECT binding or ambiguous structural changes, other nuclear imaging modalities, such as ^123^IBZM and brain perfusion SPECT, could be utilized to detect a pattern combination of different radiotracers [38,41]. Next, imaging findings could be cross-validated with fluid biomarkers. Combining DaT-SPECT metrics with CSF NfL may increase diagnostic accuracy [46].

The integration of multiple imaging modalities and laboratory-based biomarkers represents an increasingly important strategy for capturing the complex and multifactorial pathophysiological processes underlying PSP and related neurodegenerative disorders. Recent advances in multimodal data fusion approaches have demonstrated the potential of integrating neuroimaging findings with systemic biological markers, such as proteomics, to better elucidate interconnected brain–body pathways in neuropsychiatric and neurodegenerative conditions [82]. Such integrative frameworks may improve disease characterization, enhance diagnostic accuracy, facilitate patient stratification, and support the development of personalized therapeutic approaches. Future studies should therefore prioritize standardized multimodal biomarker integration strategies capable of combining imaging-derived and laboratory-derived information into unified and clinically applicable diagnostic models.

**Figure 2:**
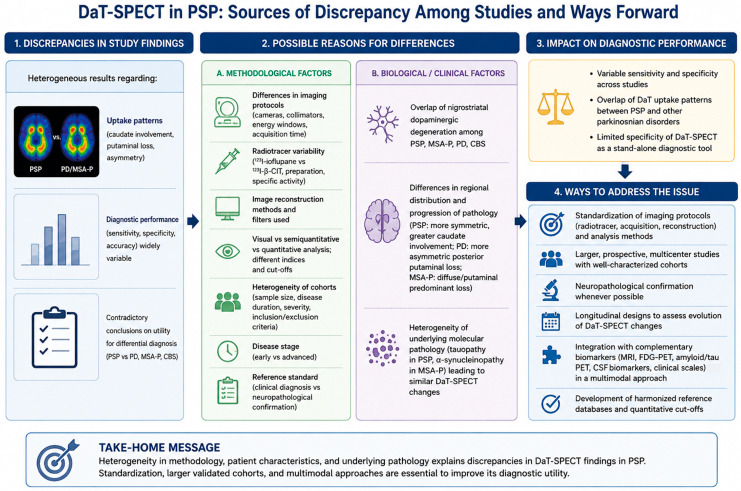
Potential reasons and solutions for the studies’ discrepancies.

Regarding patient enrollment in clinical trials, DaT-SPECT may be deployed as a biomarker for entry enrichment and cohort stratification rather than a primary outcome measure. Thus, baseline DaT-SPECT could confirm objective nigrostriatal degeneration, successfully excluding misdiagnosed cases [83]. Moreover, due to the inherent heterogeneity across PSP variants, prospective studies tracking longitudinal DaT-SPECT decline as a secondary endpoint require rigorous power calculations. Finally, the critical limitation of existing literature remains the lack of neuropathological confirmation. Future prospective trials must embed mandatory post-mortem brain donation protocols. Validating early-stage DaT-SPECT profiles against quantitative post-mortem tau burden and nigral neuronal loss is the only definitive path to establishing true sensitivity and specificity thresholds for this imaging modality. Figure 2 summarizes discrepancies among the results of different studies, possible reasons for these differences, and potential ways to address them.

## 5. Study Limitations

We acknowledge several limitations in our study. First, this work is structured as a narrative review rather than a systematic review or meta-analysis; thus, a formal quantitative or qualitative data synthesis was not performed. Instead, our primary objective was to discuss the potential clinical utility of DaT-SPECT in PSP across diagnostic, prognostic, and disease surveillance frameworks. Second, our literature search was restricted to the PubMed database. Consequently, some relevant original research articles may have been omitted. Moreover, a standardized tool for formal quality assessment, risk of bias evaluation, or the appraisal of methodological rigor was not utilized. This is particularly relevant regarding the diagnostic performance metrics, such as sensitivity and specificity, reported across the included studies. While not an exhaustive bibliographic mapping, this targeted approach allowed us to effectively highlight the core clinical applications, inherent limitations, and future directions of DaT-SPECT imaging in progressive supranuclear palsy. Regardless, both the small number and the heterogeneity of the included studies limit the generalizability of our conclusions. Future systematic research utilizing a formal methodological analysis would provide highly valuable and complementary insights to our current narrative overview.

## 6. Conclusions

In conclusion, DaT-SPECT appears to play a role in the diagnosis and overall evaluation of disease progression and underlying pathobiological processes in PSP. However, it is unlikely to serve as a reliable standalone diagnostic tool. Combining DaT-SPECT with other diagnostic biomarkers—particularly the MRPI derived from MRI, as well as other nuclear imaging modalities such as brain perfusion SPECT—may improve diagnostic accuracy, especially given its relatively low specificity for PSP. Nevertheless, these initially promising findings need to be validated in large, multicenter studies that include and clearly define multiple PSP subtypes. Ideally, such studies should involve cases that are ultimately confirmed by autopsy, given the diagnostic uncertainty during life and the potential for overlapping pathologies.

## Figures and Tables

**Table 1:** Key characteristics of studies using DaT-SPECT ^1^ as the sole imaging modality in patients with PSP ^2^.

Study	PSP Patients (n)	Other Study Groups (n)	Additional Modalities	Main Findings
Parkinson Study Group, 2000 [21]	17	PD ^3^ (43), ET ^4^ (14), NC ^5^ (22)	-	-0.98 sensitivity and 0.83 specificity for discriminating between PD and PSP patients to ET and NC
Seppi et al., 2006 [22]	14	PD (17), MSA-P ^6^ (15), NC (13)	-	-reduced basal ganglia uptake for all patient groups compared to controls -reduced midbrain uptake for PSP and MSA-P compared to PD
Goebel et al., 2011 [23]	15	PD (15), MSA-P (15), NC (15)	MRI ^7^	-83.3% accuracy in diagnosing PSP, PD, and MSA-P (93.3% if PSP and MSA-P combined)
Im et al., 2006 [24]	9	PD (20), NC (10)	-	-greater uptake reduction in ROI ^8^ between caudate and putamen for PSP (72%) compared to PD (53%) -different reduction uptake patterns between PSP and PD
Antonini et al., 2002 [25]	10	PD (70), MSA-P (10), NC (12)	-	-less uptake in PSP compared to PD and MSA-P
Filippi et al., 2006 [16]	15	PD (21), NC (20)	-	-reduced uptake in PSP and PD compared to NC -reduced uptake in PSP compared to PD -asymmetrical uptake in PD and PSP
Roselli et al., 2010 [26]	8	PD (15), DLB ^9^ (15), ET (15), NC (9)	MRI	-severely decreased serotonin transporter uptake in midbrain for PSP and DLB -mildly decreased for PD compared to ET and NC
Joling et al., 2017 [27]	13	PD (30), MSA-P (9), MSA-C ^10^ (7)	MRI	-lower tracer uptake in the caudate nucleus for PSP compared to PD and MSA-C -lower tracer uptake in the hypothalamus for PSP compared to MSA-C
Badoud et al., 2016 [28]	32	PD (306), MSA ^11^ (24), CBD ^12^ (30)	MRI	-greater uptake reduction in the head of the caudate for PSP and MSA compared to PD and CBD -0.69 AUC ^13^ for PSP against MSA and CBD -0.69 AUC for PD against PSP, MSA, and CBD combined
Ueda et al., 2017 [19]	8	PD (50), DLB (21), MSA (5), CBD (7), ET (7), NPH ^14^ (2), VaP ^15^ (3), DIP ^16^ (11)	-	-91.2% sensitivity with visual assessment for differentiating neurodegenerative Parkinsonism -86.8% sensitivity with semi-quantitative assessment -96.7% sensitivity with combined visual and semi-quantitative assessment
Sakamoto et al., 2020 [29]	20	PD (11), MSA (8), CBD (4), other (10)	MRI	-95.0% sensitivity, 36.4% specificity, 58.5% accuracy with SBR ^17^ -85.0% sensitivity, 100% specificity, 94.3% accuracy with MRPI ^18^
Seckin et al., 2020 [30]	15	CBD (8), PPAOS ^19^ (17)	-	-lower uptake in the posterior putamen in the PSP group
Shigekiyo et al., 2020 [31]	33	PD (311), MSA-P (20), NC (137)	-	-higher average SBR and SBR laterality in PD HY ^20^ stage I compared to PSP
Constantinides et al., 2023 [32]	42	PD (17), MSA-P (15), CBD (28), NC (35)	-	-74.1% sensitivity and 85.0% specificity for mean SBR differences between PSP and CBD -insignificant differences between uptake in PSP, PD, and MSA-P
Ishizawa et al., 2023 [33]	45	PD (122), PDD ^21^ (19), DLB (30), MSA-P (18), NC (18)	MRI	-striatal uptake did not differ among PSP, DLB, and MSA-P -reduced uptake in the posterior striatum for PD and PDD
Kawazoe et al., 2024 [34]	30	MSA-P (29), CBD (31)	MRI	-insignificant SBR and MRI differences between PSP and CBD -no correlations between SBR and MRI parameters among all study groups
Hastings et al., 2024 [35]	10	PD (47), MSA (42), CBD (10)	autopsy	-93.2% sensitivity, 52.9% specificity, and 72.2% NPV ^22^ for PSP

^1^ DaT-SPECT: dopamine transporter single-photon emission computed tomography, ^2^ PSP: progressive supranuclear palsy, ^3^ PD: Parkinson’s disease, ^4^ ET: essential tremor, ^5^ NC: normal controls, ^6^ MSA-P: multiple system atrophy with predominant Parkinsonism, ^7^ MRI: magnetic resonance imaging, ^8^ ROI: region of interest, ^9^ DLB: dementia with Lewy bodies, ^10^ MSA-C: multiple system atrophy-cerebellar type, ^11^ MSA: multiple system atrophy, ^12^ CBD: corticobasal degeneration, ^13^ AUC: area under the curve, ^14^ NPH: normal pressure hydrocephalus, ^15^ VaP: vascular parkinsonism, ^16^ DIP: drug-induced parkinsonism, ^17^ SBR: specific binding ratio, ^18^ MRPI: magnetic resonance parkinsonism index, ^19^ PPAOS: primary progressive apraxia of speech, ^20^ HY: Hoehn and Yahr scale, ^21^ PDD: PD dementia, ^22^ NPV: negative predictive value.

**Table 2:** Key characteristics of studies using DaT-SPECT ^1^ in combination with additional molecular imaging modalities in patients with PSP ^2^.

Study	PSP Patients (n)	Other Study Groups (n)	Additional Modalities	Main Findings
Kim et al., 2002 [36]	6	PD ^3^ (18), MSA ^4^ (7), NC ^5^ (29)	^123^I-IBF ^6^, MRI ^7^	-reduced DaT uptake in the posterior putamen for all patient groups -higher caudate D2 receptor uptake for PD and PSP, compared to MSA
Plotkin et al., 2005 [37]	8	PD (25), MSA (13), DLB ^8^ (6), CBD ^9^ (9), ET ^10^ (11)	^123^I-IBZM ^11^	-reduced ^123^I-IBZM uptake in PSP and MSA -normal ^123^I-IBZM uptake in PD, ET, and the majority of CBD patients -reduced DaT uptake in all Parkinsonian disorders
Lin et al., 2010 [38]	PSP-RS ^12^ (6), PSP-P ^13^ (4)	PD (10), NC (7)	^123^I-IBZM	-insignificantly higher DaT uptake in PSP-RS compared with PSP-P -increased caudate-to-putamen uptake ratio in PD, decreased in PSP -reduced IBZM uptake in PSP-RS
Jakobson et al., 2013 [39]	16	PD (29), MSA (12), NC (16)	^123^I-IBZM	-lower striatal DaT initial uptake for PSP compared to MSA, similar uptake levels after three years -higher ^123^I-IBZM uptake for MSA compared with PSP initially and to all study groups subsequently
Van Laere et al., 2006 [18]	12	PD (58), MSA (24), DLB (8), ET (27)	^99m^Tc-ECD ^14^	-lower DaT uptake for PSP compared to PD, similar to DLB and MSA -different anterior-to-posterior putamen ratio of the left side for PSP compared to PD and MSA patients -58.8% diagnostic accuracy with DaT (higher for PSP specifically) -−67.6% diagnostic accuracy with brain perfusion SPECT -82.4% diagnostic accuracy with both DaT-SPECT and brain perfusion SPECT
Takaya et al., 2018 [40]	10	PD (44), MSA (15), DLB (2), CBD (8)	^123^I-IMP ^15^, MRI	-0.978 AUC ^16^ in discriminating PSP from PD and DLB -0.920 AUC in discriminating PSP from MSA -0.875 AUC in discriminating PSP from CBD
Nakano et al., 2022 [41]	PSP-RS (14), PSP-PGF ^18^ (4), PSP-PI ^19^ (2), PSP-SL ^20^ (1)	PD (58), MSA (24), CBD (26)	^123^I-IMP	-0.80 AUC in discriminating PSP from PD -correlation of ipsilateral DaT SBR ^17^ with basal ganglia ipsilateral prefrontal cortex perfusion for the PSP-RS subgroup
Niimi et al., 2017 [42]	3	PD (13), MSA (4), CBD (5), other (7)	^123^I-MIBG ^21^	-normal cardiac ^123^I-MIBG in all patients (prerequisite) -similar SBR among all study groups -lower SAI ^22^ in PD patients compared to other study groups
Iwabuchi et al., 2021 [43]	16	PD (90), MSA (9), DLB (21), NC (80)	^123^I-MIBG	-50.0% sensitivity, 98.0% specificity, 96.1% NPV for PSP

^1^ DaT-SPECT: dopamine transporter single-photon emission computed tomography, ^2^ PSP: progressive supranuclear palsy, ^3^ PD: Parkinson’s disease, ^4^ MSA: multiple system atrophy, ^5^ NC: normal controls, ^6 123^I-IBF: ^123^I iodobenzofuran, ^7^ MRI: magnetic resonance imaging, ^8^ DLB: dementia with Lewy bodies, ^9^ CBD: corticobasal degeneration, ^10^ ET: essential tremor, ^11 123^I-IBZM: ^123^I-iodobenzamide, ^12^ PSP-RS: PSP-Richardson syndrome, ^13^ PSP-P: PSP with predominant Parkinsonism, ^14 99m^Tc-ECD: ^99m^Tc ethylcysteinate dimer, ^15 123^I-IMP: ^123^I iodoamphetamine, ^16^ AUC: area under the curve, ^17^ SBR: specific binding ratio, ^18^ PSP-PGF: PSP with progressive gait freezing, ^19^ PSP-PI: PSP with postural instability, ^20^ PSP-SL: PSP with speech and language impairment, ^21 123^I-MIBG: ^123^I-metaiodobenzylguanidine, ^22^ SAI: striatal asymmetry index.

**Table 3:** Summary of studies combining DaT-SPECT ^1^ with laboratory biomarkers in PSP ^2^.

Study	PSP Patients (n)	Other Study Groups (n)	Additional Modalities	Main Findings
Suzuki et al., 2019 [45]	15	PD ^3^ (73), MSA ^4^ (22), CBD ^5^ (2), DLB ^6^ (14), AD ^7^ (18), ALS ^8^ (6), FTD ^9^ (6)	serum IGF-1 ^10^	-similar IGF-1 levels among all study groups -insignificant SBR ^11^ DaT-SPECT differences among all Parkinsonian syndromes
Diekämper et al., 2021 [46]	12	PD (10), DLB (7), AD (17), CBD (1), NC ^12^ (13)	CSF ^13^ NfL ^14^, pNfH ^15^, α-synuclein, total tau, progranulin	-negative correlation of bilateral caudate nucleus and putamen uptake and CSF levels of NfL and pNfH for PSP, PD, CBD, and DLB -insignificant differences in DaT uptake and NfL and pNfH between PSP, PD, CBD, and DLB
Goto et al., 2023 [47]	12	PD (70), MSA-P ^16^ (5), MSA-C ^17^ (7), CBD (6), AD (9)	CSF HVA ^18^, autopsy (for two PSP patients)	-lower SBR for PSP compared to PD -lower CSF HVA in PSP, PD, and MSA compared to AD -positive correlation between SBR in DaT-SPECT and CSF HVA for PSP and PD patients
Shimada et al., 2025 [48]	PSP-RS ^19^ (5), PSP-P ^20^ (5), PSP-PGF ^21^ (5)	NPH ^22^ (22)	^123^I-IMP-SPECT ^23^, CSF α-synuclein, MRI ^24^	-reduced SBR was reduced in PSP plus NPH compared to NPH-only group -asymmetrically reduced DaT uptake in PSP plus NPH—clinical improvement in patients with PSP plus NPH one year after lumboperitoneal shunt
Ozawa et al., 2024 [49]	19	PD (62), MSA (25)	^123^I-MIBG ^25^, SSR ^26^	-correlation of DaT-SPECT and ^123^I-MIBG uptake with SSR amplitude only in the PD group

^1^ DaT-SPECT: dopamine transporter single-photon emission computed tomography, ^2^ PSP: progressive supranuclear palsy, ^3^ PD: Parkinson’s disease, ^4^ MSA: multiple system atrophy, ^5^ CBD: corticobasal degeneration, ^6^ DLB: dementia with Lewy bodies, ^7^ AD: Alzheimer’s disease, ^8^ ALS: amyotrophic lateral sclerosis, ^9^ FTD: frontotemporal dementia, ^10^ IGF-1: insulin-like growth factor-1, ^11^ SBR: specific binding ratio, ^12^ NC: normal controls, ^13^ CSF: cerebrospinal fluid, ^14^ NfL: neurofilament light chain, ^15^ pNfH: phosphorylated neurofilament heavy chain, ^16^ MSA-P: multiple system atrophy with predominant Parkinsonism, ^17^ MSA-C: multiple system atrophy-cerebellar type, ^18^ HVA: homovanillic acid, ^19^ PSP-RS: PSP-Richardson syndrome, ^20^ PSP-P: PSP with predominant parkinsonism, ^21^ PSP-PGF: PSP with progressive gait freezing, ^22^ NPH: normal pressure hydrocephalus, ^23 123^I-IMP: ^123^I iodoamphetamine single-photon emission computed tomography, ^24^ MRI: magnetic resonance imaging, ^25 123^I-MIBG: ^123^I-metaiodobenzylguanidine, ^26^ SSR: sympathetic skin response.

## Data Availability

No new data were generated during the preparation of this manuscript.

## Abbreviations

The following abbreviations are used in this manuscript:

^123^I-β-CIT	^123^I 2β-carboxymethoxy-3β-(4-iodophenyl)tropane
^123^I-IBF	^123^I iodobenzofuran
^123^I-IBZM	^123^I iodobenzamide
^123^I-FP-CIT	^123^I N-ω-fluoropropyl-2β-carbomethoxy-3β-(4-iodophenyl)nortropane
^123^I-IPT	^123^I N-(3-iodopropen-2-yl)-2β-carbomethoxy-3β-(4-chlorophenyl)tropane
^123^I-MIBG	^123^I metaiodobenzylguanidine
^123^I-IMP	^123^I iodoamphetamine
4R-tau	four-repeat tau isoforms
^99m^Tc-ECD	^99m^Tc ethylcysteinate dimer
^99m^Tc-TRODAT-1	[2-[[2-[[[3-(4-chlorophenyl)-8-methyl-8-azabicyclo [3,2,1]oct-2-yl]methyl](2-mercaptoethyl)amino]ethyl]amino]ethanethiolato(3-)-N2,N20,S2,S20]oxo [1R-(exo-exo)])-[^99m^Tc] dopamine transporter
AD	Alzheimer’s disease
ALS	amyotrophic lateral sclerosis
APD	atypical Parkinsonian disorders
AUC	area under the curve
CBD	corticobasal degeneration
CSF	cerebrospinal fluid
DaT-SPECT	dopamine transporter single-photon emission computed tomography
DIP	drug-induced Parkinsonism
DLB	dementia with Lewy bodies
ET	essential tremor
FTD	frontotemporal dementia
FDG-PET	fluorodeoxyglucose positron emission tomography
DaT-PET	dopamine transporter positron emission tomography
HVA	homovanillic acid
HY	Hoehn and Yahr Scale
IGF-1	insulin-like growth factor-1
NfL	neurofilament light chain
NPV	negative predictive value
MAPT	microtubule-associated protein tau
MDS	Movement Disorders Society
MRI	magnetic resonance imaging
MRPI	magnetic resonance Parkinsonism index
MSA	multiple system atrophy
MSA-C	MSA-cerebellar type
MSA-P	MSA with predominant Parkinsonism
NINDS	National Institute of Neurological Disorders and Stroke
NPH	normal pressure hydrocephalus
PD	Parkinson’s disease
PDD	PD dementia
pNfH	phosphorylated neurofilament heavy chain
PPAOS	primary progressive apraxia of speech
PSP	progressive supranuclear palsy
PSP-P	PSP with predominant Parkinsonism
PSP-PGF	PSP with progressive gait freezing
PSP-PI	PSP with postural instability
PSP-RS	PSP-Richardson syndrome
PSP-SL	PSP with speech and language impairment
ROI	region of interest
SAI	striatal asymmetry index
SANRA	scale for the quality assessment of narrative review articles
SBR	specific binding ratio
SPECT	single-photon emission computed tomography
SSR	sympathetic skin response
VaP	vascular Parkinsonism
